# The Relationship Between Medical Student Neuroticism and Preferred Medical Specialty: Cross-Sectional Study

**DOI:** 10.2196/73507

**Published:** 2025-06-18

**Authors:** Jonathan Shaw, Brenton Phung, Ashley Lai, James Hagerty, Van Le, Seung Rim Yoo, Jerome Torres, Angelene Won, Charles Lai, Peter Bota, Aaron Jacobs

**Affiliations:** 1 School of Medicine, California University of Science and Medicine, 1501 Violet St, Colton, CA, 92324, United States, 1 9094980036; 2 Medical Education, California University of Science and Medicine, Colton, CA, United States

**Keywords:** neuroticism, medical students, preclinical, specialty interest, medical education

## Introduction

Choosing a specialty is a key decision for US medical students, finalized in the fourth year through the National Resident Matching Program. Influencing factors include experience, gender, lifestyle, finances, and personality [1]. Aligning personality with specialty can boost job satisfaction and patient care [2]. Further, 51% of preclerkship students eye competitive fields, making early insights valuable [3,4].

The 5-factor model includes extraversion, agreeableness, openness, conscientiousness, and neuroticism [5,6]. Neuroticism—linked to anxiety and emotional instability—affects decision-making [7], but its role in specialty choice is underexplored [2]. Studies show that surgeons tend to have low neuroticism [6,8], while future obstetricians and psychiatrists show higher levels [9,10].

This study examines the relationship between neuroticism and specialty choice among medical students at a US allopathic medical school. The goal is to provide insights for personalized medical career counseling and interventions tailored to students’ needs.

## Methods

### Participants and Recruitment

First-year and second-year medical students (n=120 and n=126, respectively) from a California allopathic school with a pass/fail curriculum were invited to complete an anonymous survey from January 11 to 25, 2024, via institutional email. A convenience sample of 32 responses (22 and 10 responses from first-year and second-year medical students, respectively) was collected.

### Measures

The survey included demographic questions (school year and gender), 8 questions from the Big Five Inventory assessing neuroticism, and specialty-related questions (specialty interests, perceived competitiveness of chosen specialties, and self-perceived preparedness for matching). Question order was randomized.

### Statistical Analysis

Data analysis was conducted using IBM SPSS Statistics 28.0.1.0 (IBM Corp). Normality was assessed via Kolmogorov-Smirnov tests. Independent samples *t* tests (2-tailed) were used for normal data; Kruskal-Wallis tests were used for nonnormal data. The grouping variables were school year, gender, and specialty interest type. Statistical significance was determined as *P*≤.05.

### Ethical Considerations

This study received ethical approval from the California University of Science and Medicine Institutional Review Board (approval: HS-2023‐59) on November 27, 2023. Informed consent for primary data collection and secondary analyses of the research data was obtained from all individual participants included in this study. Participants received no compensation for participation.

## Results

Kolmogorov-Smirnov tests indicated normal distributions for self-reported surgical, medical, and overall competitiveness, as well as neuroticism scores. Independent *t* tests revealed no significant differences based on gender, school year, or specialty interest type. However, students interested in both surgical and medical specialties had lower neuroticism scores (*t*_30_=−2.039; *P*=.03) than those of students who focused on only one field.

Second-year medical students reported feeling tenser (H_1_=4.867; *P*=.03) and moodier (H_1_=10.364; *P*=.001) than first-year medical students. Students interested in surgical specialties perceived their specialties as more competitive (H_1_=4.080; *P*=.04). Participants interested in both surgery and medicine reported feeling more relaxed (H_1_=5.882; *P*=.02) than those interested in only one field. Further survey data are presented in Table 1.

**Table 1.** Descriptive statistics for survey items from this cross-sectional, web-based survey study, which was conducted at a California allopathic medical school via institutional email from January 11 to 25, 2024.

Survey items	Responses, n	Minimum score	Maximum score	Score, mean	Score, SD
Which school year are you?^a^	32	1	2	1.31	0.471
What gender do you identify as?^b^	32	1	2	1.41	0.499
Surgery^c^	32	1	2	1.59	0.499
Medicine^c^	32	1	2	1.19	0.397
Both^c^	32	1	2	1.69	0.471
Surgical competitiveness^d^	15	1	5	3.27	1.123
Medical competitiveness^d^	25	1	5	2.83	1.197
Overall competitiveness^d^	29	1	5	2.91	1.058
Neuroticism vs emotional stability^e^	32	6	26	15.19	6.061
Is depressed, blue^f^	32	1	4	2.25	1.218
Is relaxed, handles stress well^f^	32	1	5	3.72	1.301
Can be tense^f^	32	1	5	3.34	1.153
Worries a lot^f^	32	1	5	3.28	1.35
Is emotionally stable, not easily upset^f^	32	1	5	4.03	1.15
Can be moody^f^	32	1	5	2.84	1.247
Remains calm in tense situations^f^	32	2	5	3.84	1.11
Gets nervous easily^f^	32	1	5	3.34	1.181
Medical school feels overwhelming^f^	32	1	5	3.16	1.37
I feel I am very prepared academically^f^	32	1	5	3.5	1.164
I am a competitive applicant for the specialties I have in mind^f^	32	1	5	3.44	1.216
I worry a lot about matching into my preferred specialty^f^	32	1	5	3.25	1.27
I am confident in my ability to succeed as a medical student^f^	32	1	5	3.91	1.027
The specialties I am considering are not that hard to match into^f^	32	1	5	2.78	1.289
I have a plan for how I’m going to match into the specialty that I want^f^	32	1	5	3.5	1.164
I have access to enough resources to become a competitive applicant for my specialties of interest^f^	32	1	5	3.69	1.176
I’m worried about not having enough research/publications for my specialty of interest^f^	32	1	5	3.66	1.335
I’ll have to take a gap/research year to match into my specialties of choice^f^	32	1	5	1.84	1.194

a 1=first year; 2=second year.

b 1=woman; 2=man.

c Participants’ interest in surgical, medical, and mixed/both specialties was indicated in their demographic section by checking either one or both types of specialties (1=no; 2=yes).

d Competitiveness was determined by participants self-reporting their perceived competitiveness on a scale of 1 to 5, with 1 being “not very competitive,” 3 being “average competitiveness,” and 5 being “very competitive.” Participants indicated which specific specialties they wished to pursue (urology, emergency medicine, etc), and these were used to determine which group (surgical vs medical) their responses were analyzed with.

e Neuroticism vs emotional stability was determined by summing participant responses to a series of 5-point Likert questions from the 5-factor personality test, with 1 being “disagree strongly” and 5 being “agree strongly.” Participants scoring 6 were seen as more emotionally stable, while those scoring 26 were seen as more neurotic.

f Scored on a Likert scale from 1 to 5, with 1 being “I disagree strongly” and 5 being “I agree strongly.”

## Discussion

We found that surgery-interested students viewed their field as more competitive, but their neuroticism levels were similar to those of peers interested in other fields. They may need more academic support, and mental health resources should be cohort-specific, as participants showed moderately elevated neuroticism (mean 15.19, SD 6.061) when compared to literature [6].

Surgery-interested students viewed fields like orthopedics and neurosurgery as more competitive, which is consistent with prior research [2]. Unlike existing literature, this study found similar neuroticism levels between surgical and medical students (*F*_30_=0.220; *P*=.64) [9,10]. Students interested in both surgical and medical specialties reported lower neuroticism and better stress management (*F*_30_=5.142; *P*=.03), possibly due to having interest in less competitive fields or having backup plans. No gender differences were found, though second-year medical students showed higher tension (H_1_=4.867; *P*=.03) and moodiness (H_1_=10.364; *P*=.001), likely due to board examination stress. The absence of gender effects may be due to the small sample size of 32 participants.

These discrepancies may have resulted from the small sample size or the early stage of participants’ medical education. Since this study was not longitudinal, it could not capture changes in specialty interests that may occur during participants’ clinical years. Although the medical program’s pass/fail curriculum may have helped with reducing stress and neuroticism, its impact remains unclear due to this study’s single-institution scope.

This study found a link between neuroticism and specialty interest among medical students. The pressure to choose and prepare for a specialty so early in medical school may explain the neuroticism and stress found in preclinical participants, highlighting the need for early experiences with specialties, so that students can select their field and begin tailoring their activities as early as possible.
